# Identification of Anxiety and Depression Symptoms in Patients With Cancer: Comparison Between Short and Long Web-Based Questionnaires

**DOI:** 10.2196/11387

**Published:** 2019-04-05

**Authors:** Susanne Mattsson, Erik Martin Gustaf Olsson, Maria Carlsson, Birgitta Beda Kristina Johansson

**Affiliations:** 1 Lifestyle and Rehabilitation in long term illness Public Health and Caring Sciences Uppsala University Uppsala Sweden; 2 Clinical Psychology in Healthcare Department of Women's and Children's Health Uppsala University Uppsala Sweden; 3 Experimental and Clinical Oncology Department of immunology, genetics and pathology Uppsala University Uppsala Sweden

**Keywords:** screening, cancer, depression, anxiety, internet, eHealth

## Abstract

**Background:**

Physicians and nurses in cancer care easily fail to detect symptoms of psychological distress because of barriers such as lack of time, training on screening methods, and knowledge about how to diagnose anxiety and depression. National guidelines in several countries recommend routine screening for emotional distress in patients with cancer, but in many clinics, this is not implemented. By inventing screening methods that are time-efficient, such as digitalized and automatized screenings with short instruments, we can alleviate the burden on patients and staff.

**Objective:**

The aim of this study was to compare Web-based versions of the ultrashort electronic Visual Analogue Scale (eVAS) anxiety and eVAS depression and the short Hospital Anxiety and Depression Scale (HADS) with Web-based versions of the longer Montgomery Åsberg Depression Rating Scale-Self-report (MADRS-S) and the State Trait Anxiety Inventory- State (STAI-S) with regard to their ability to identify symptoms of anxiety and depression in patients with cancer.

**Methods:**

Data were obtained from a consecutive sample of patients with newly diagnosed (<6 months) breast, prostate, or colorectal cancer or with recurrence of colorectal cancer (N=558). The patients were recruited at 4 hospitals in Sweden between April 2013 and September 2015, as part of an intervention study administered via the internet. All questionnaires were completed on the Web at the baseline assessment in the intervention study.

**Results:**

The ultrashort and short Web-based-delivered eVAS anxiety, eVAS depression and HADS were found to have an excellent ability to discriminate between persons with and without clinical levels of symptoms of anxiety and depression compared with recommended cutoffs of the longer instruments MADRS-S and STAI-S (area under the curve: 0.88-0.94). Cutoffs of >6 on HADS anxiety and >7 hundredths (hs) on eVAS anxiety identified patients with anxiety symptoms with high accuracy. For HADS depression, at a cutoff of >5 and eVAS depression at a cutoff of >7 hs, the accuracy was very high likewise.

**Conclusions:**

The use of the short and ultrashort tools, eVAS and HADS, may be a suitable initial method of Web-based screening in busy clinical settings. However, there are still a proportion of patients who lack access to the internet or the ability to use it. There is a need to find solutions for this group to find all the patients with psychological distress.

## Introduction

Symptoms of anxiety and depression are common in people diagnosed with cancer depending on the specific diagnosis, stage of the disease, sex, age, and the employed screening instrument [1,2]. According to a large Canadian study (n=10,153), almost 1 of 5 patients experienced clinical anxiety symptoms and clinical depression symptoms were present in about 12.87% (1204/9357) of the patients [1]. We found that 35.6% (176/495) of patients recently referred to the oncology department (<1 month) because of cancer disease experienced anxiety or depression symptoms and that more than 1 of 5 experienced such symptoms 6 months later [3]. Anxiety and depression in patients with cancer are associated with poor health-related quality of life, disease-related morbidity, poor treatment adherence, and prognosis [3-8].

Physicians and nurses in cancer care easily fail to detect symptoms of psychological distress because of lack of time and knowledge about how to diagnose anxiety and depression [9]. Consequently, many patients have an unmet need for psychosocial support [10]. Therefore, national guidelines in several countries recommend routine screening for emotional distress in patients with cancer [8,11-13]. On the contrary, routine clinical screening is still controversial and partly questioned because studies have yielded ambiguous results [14-16]. Our previous research project concluded that systematic screening for anxiety and depression is feasible in a clinical oncological setting and increases referral for clinical assessment, psychosocial support, and treatment [3]. However, the implementation of screening in routine care, without additional research funding, places significant challenges on an already strained cancer care. Lack of resources and absence of screening strategies, as well as receipt of appropriate aftercare, have been defined as barriers for a successful implementation of screening [2,9].

Screening for anxiety and depression using validated questionnaires is a standard procedure in research. Commonly used questionnaires vary considerably with regard to the number of items [16]. Health professionals prefer shorter questionnaires as they are less time-consuming than longer ones [9]. Ultrashort questionnaires (1 to 4 items) may be as successful as short questionnaires (5 to 20 items), hence efficient and acceptable, and are therefore suggested to be suitable for initial assessment in clinical settings [17]. Long questionnaires generally have a higher specificity, but as the clinical acceptance is low because of the higher demand on patients and staff, they are not always useful in routine cancer care [18]. Findings from research projects evaluating Web-based screening followed by psychosocial support, when distress is identified, indicate that this may be an efficient way to reduce suffering [19-21]. Thus, Web-based screening of anxiety and depression with automatic calculations of scores can be a realistic alternative to paper-based screening in a busy cancer clinic and may be one way to increase availability and referral for support and treatment when needed. An important first step is to evaluate the accuracy of Web-based versions of clinically relevant questionnaires for screening of anxiety and depression symptoms.

Digital versions of self-report symptom scales have shown high interformat reliability compared with pen and paper versions [22]. However, the researchers concluded that these findings could not be generalized to all questionnaires and settings and that future studies regarding the evaluation of Web-based versions of traditional paper-based questionnaires should include large sample sizes. In addition, further evaluations are needed because of the novelty of the procedure of Web-based screening.

In this study, we aimed to evaluate the accuracy of the Web-based versions of the ultrashort electronic Visual Analogue Scale (eVAS) and the short Hospital Anxiety and Depression Scale (HADS) [23,24] using the Web-based versions of the longer Montgomery-Åsberg Depression Rating Scale-Self-assessment (MADRS-S) [25] and the State-Trait Anxiety Inventory-State (STAI-S) [26] as references.

## Methods

### Participants and Procedures

A consecutive sample of patients with newly diagnosed (<6 months) breast, prostate, or colorectal cancer or with recurrence of colorectal cancer were recruited at 4 hospitals in Sweden between April 2013 and September 2015, as part of an intervention study administered via the internet [27]. The exclusion criteria were inability to read and understand Swedish, cognitive disability (eg, dementia or psychosis), a constant need for hospital care (Karnofsky score <40) or short expected survival (<3 months). The participants were approached and informed by a research assistant at a regular visit to the clinic and gave their written informed consent. This study was approved by the Regional Ethical Review Board in Uppsala (Dnr. 2012/003).

### Data Collection

#### Quality Register

Background data regarding the disease and treatment were obtained from the diagnosis-specific quality registers of the Uppsala–Örebro region in Central Sweden.

#### Questionnaires

All questionnaires were completed on the Web at the baseline assessment in the intervention study [27], in a place of the participant’s choice. Demographic information (age, sex, marital status, and education) was obtained via project-specific questions.

eVAS measures anxiety and depression on 2 scales ranging from 1 to 100 hundredths (hs), that is, independent of the screen size. The paper-based VAS is evaluated to perform well when correlated to standardized measures of anxiety and depression [23]. We chose separate scales for anxiety and depression instead of a single distress scale (eg, the Distress Thermometer) [28] for a more accurate comparison with MADRS-S and STAI-S. The patients were asked to grade their levels of anxiety and depression by making a mark somewhere between 0 (no anxiety or depression) and 100 (extreme anxiety or depression) on each scale. Web-based screening with the similar ultrashort Distress Thermometer has been successfully used in a large sample of cancer patients [29].

The HADS consists of 7 questions measuring depression and 7 measuring anxiety [24] and is the most validated and widely used questionnaire in screening for anxiety and depression in patients with cancer [30]. The patients were asked to rate their emotional status during the past week on a scale of 0 to 3. The total score of each scale is 21 points. A higher score implies more symptoms, 8 to 10 points indicate doubtful cases and 11 to 21 points indicate clinically significant cases of anxiety or depression, according to Zigmond and Snaith [24]. However, further evaluations of the HADS have revealed large variations in recommended cutoff scores for depression and anxiety in various groups of patients with cancer, both higher and lower scores have been suggested as cutoffs for clinically significant symptoms [31-33]. Thus, the optimal cutoff scores for identification of patients with a need for support are still not decided. Screening with computerized versions of the HADS in patients with cancer has been deemed feasible [34]. In addition, a comparison between a Web-based-delivered version and a paper version of HADS has yielded comparable findings with regard to frequencies and intensity of symptoms [35].

The STAI-S was used as a reference to eVAS and HADS anxiety. STAI-S is a self-administered questionnaire that measures participants’ state of anxiety [26]. It has been used in patients with various cancer diagnoses and administered on the Web [36]. STAI-S comprises 20 items where the respondents are asked to rate their current feelings on a 4-point scale, from *not at all* (1) *to very much* (4). The scores are summed, ranging from 20 to 80 [26]. A cutoff level of >39 was used to indicate clinically significant symptoms of anxiety in this study based on previous research in similar populations [37,38].

The MADRS-S, the self-rating version of the MADRS [25], was used as a reference to eVAS and HADS depression. The MADRS-S has acceptable psychometric properties with regard to reliability, validity, and sensitivity to change [39]. In addition, it can be transferred to the Web without affecting the psychometric properties in a clinically significant way [40]. The MADRS-S has 9 items [41], and the respondents are asked to rate their emotional status during the previous 3 days. All items range from 0 to 6, and the total score is 54. A higher value indicates more depressive symptoms. The cutoff score for depression varies greatly in studies, but the thresholds recommended by Svanborg and Ekselius (0-12=minimal, 13-19=mild, 20-34=moderate, and >34=severe) [41] have been used in previous Swedish studies where the instrument was administered both on the Web and in paper and pen versions [42-44]. A cutoff of >12 has been applied in this study to indicate clinically significant symptoms of depression [41].

### Data Analysis

The statistical analyses were performed using IBM Statistical Package for the Social Sciences (version 20.0) [45]. Receiver operating curve (ROC) analyses were used to assess the sensitivity and specificity of eVAS and HADS using STAI-S and MADRS-S as references. The ROCs depict the tradeoff between specificity and sensitivity for every possible cutoff score on the HADS and eVAS indexes. The area under the curve (AUC) can be interpreted as the probability that a randomly selected patient with anxiety or depression according to the STAI-S or MADRS-S, respectively, will score higher on the respective HADS and eVAS scales than a randomly selected patient without anxiety or depression according to the STAI-S or MADRS-S, respectively [46]. By calculating the AUC, it was possible to estimate the overall discriminative performance of the HADS and the eVAS with regard to the identification of patients with clinically significant levels of anxiety or depression symptoms. An AUC of 0.50 indicates a level of accuracy no better than chance and 0.70 to 0.80 represents acceptable discrimination. An AUC of ˃0.80 represents excellent discrimination, and an AUC of 1.0 indicates a test with perfect accuracy.

The specificity, sensitivity, positive predictive value (PPV), and negative predictive value (NPV) were calculated to establish relevant cutoff scores for each of the ultrashort and short instruments. Sensitivity, specificity, and NPV are the most important indicators for establishing cutoffs as the aim of the screening is to detect as many as possible and to make sure that as few as possible are false negatives.

## Results

### Participants

A total of 1748 patients were assessed for eligibility, of whom 251 were excluded and 771 declined participation mainly because of a lack of internet access or lack of interest. A total of 726 (48.5%, 726/1497) study-eligible patients were enrolled and 558 (76.9%, 558/726) of them completed the questionnaires and constituted the final sample. A majority were under treatment, for example, chemotherapy, radiotherapy, or endocrine therapy. Mean age was lower in the final study sample than in the group not included. There was also a preponderance of patients with prostate cancer and a significantly smaller group of patients with colorectal cancer among the respondents compared with not included patients (Table 1).

**Table 1:** Demographic and clinical characteristics of the patients with cancer.

Characteristics		Respondents (N=558)	Not included^a^ (N=939)	*P* value
**Sex, n (%)**				
	Male	247 (44.3)	364 (38.8)	.14
	Female	311 (55.7)	575 (61.2)	.14
Age (years), mean (range)		61 (29-86)	69 (24-99)	<.001
**Diagnosis,** **n (%)**				
	Breast cancer	272 (48.7)	469 (49.9)	.001
	Colorectal cancer	96 (17.2)	240 (25.6)	.001
	Prostate cancer	190 (34.1)	230 (24.5)	.001
**Marital status, n (%)**				
	Married or cohabiting	435 (78.0)	—^b^	—
	Living alone	24 (4.3)	—	—
	Living apart	86 (15.4)	—	—
	Missing^c^	13 (2.3)	—	—
**Education,** **n (%)**				
	Elementary school	114 (20.4)	—	—
	High school	173(31.0)	—	—
	University-level	262 (47.0)	—	—
	Missing	9 (1.6)	—	—
**Breast cancer,** **n (%)**				
	In situ	5 (1.8)	—	—
	T0 No obvious primary tumor	14 (5.1)	—	—
	T1-T4	246 (90.5)	—	—
	≥1 lymph node involved	39 (14.3)	—	—
	Distant metastases	4 (1.5)	—	—
	Surgery	258 (94.9)	—	—
	Radiation therapy	231 (84.9)	—	—
	Chemotherapy	121 (44.5)	—	—
	Endocrine therapy	191 (70.2)	—	—
	Missing^c^	7 (2.6)	—	—
**Prostate cancer,** **n (%)**				
	Unassessable tumor (TX)	2 (1.1)	—	—
	T1-T4	183 (96.3)	—	—
	≥1 lymph node involved	15 (7.9)	—	—
	Distant metastases	15 (7.9)	—	—
	Surgery	34 (17.9)	—	—
	Radiation therapy	86 (45.3)	—	—
	Chemotherapy	2 (1.1)	—	—
	Expectancy or surveillance	26 (13.7)	—	—
	Endocrine therapy	30 (15.8)	—	—
	Missing^c^	5 (2.6)	—	—
**Colorectal cancer,** **n (%)**				
	Primary tumor not found (T0)	6 (6.3)	—	—
	T1-T4	70 (72.9)	—	—
	≥1 lymph node involved	34 (35.4)	—	—
	Distant metastases	5 (5.2)	—	—
	Surgery	77 (80.2)	—	—
	Radiation therapy	22 (22.9)	—	—
	Chemotherapy	34 (35.4)	—	—
	Targeted drugs	39 (40.6)	—	—
	Missing^c^	20 (20.8)	—	—

^a^Not included: internal dropouts (n=168) and declined participation (n=771).

^b^Data not available.

^c^Missing tumor stage.

### Cases of Anxiety and Depression

The STAI-S (>39) identified 165 (30.0%, 165/550) cases and 385 noncases of anxiety, and MADRS-S (>12) identified 107 (19.4%, 107/551) cases and 444 noncases of depression. HADS anxiety identified 126 (22.6%, 126/558) participants with at least mild levels of anxiety and HADS depression identified 79 (14.2%, 79/558) participants with at least mild symptoms of depression, both scales using a cutoff score of >7. The vast majority of the scores on eVAS anxiety and depression were within the range of 1 to 20 hs (81.3%, 451/555 and 82.8%, 457/552, respectively; Table 2).

### Anxiety—Accuracy of Hospital Anxiety and Depression Scale and Electronic Visual Analogue Scale Compared With State Trait Anxiety Inventory

Using STAI-S as the reference instrument and >39 as the cutoff for clinical symptoms of anxiety, the AUC for HADS anxiety was 0.93 (95% CI 0.91-0.95). Both 5 and 6 were cutoffs with good sensitivity and specificity. A score >5 identified 95% of the patients with symptoms and ruled out 71% of those without and >6 identified 88% of the patients with symptoms and ruled out 81% of those without. The corresponding AUC for the eVAS anxiety was 0.90 (95% CI 0.87-0.93), and a cutoff score of >7 hs identified 86% of the patients with symptoms of anxiety and ruled out 83% of the patients without. The NPV values, that is, the proportion of all the undetected cases that did not have symptoms according to the reference, were generally high for the mentioned cutoffs (≥90%), whereas the PPV values, that is, the proportion of all the detected cases that actually had symptoms according to the reference, were somewhat lower (≥67%). These most important values correspond to an excellent overall accuracy (Table 3).

### Depression—Accuracy of Hospital Anxiety and Depression Scale and Electronic Visual Analogue Scale Compared With Montgomery Åsberg Depression Rating Scale-Self-Report

Using MADRS-S as the reference instrument and >12 as the cutoff for clinical symptoms of depression, the AUC for HADS depression was 0.94 (95% CI 0.92-0.96). Both 5 and 6 were cutoffs with good sensitivity and specificity. A score >5 identified 91% of the patients with symptoms and ruled out 83% of those without, and a score >6 identified 87% of those with symptoms and ruled out 89% of those without. The corresponding AUC for the eVAS depression was 0.88 (95% CI 0.84-0.93). A cutoff score of >7 hs is a cutoff with good sensitivity and specificity. It identified 85% of the patients with depressive symptoms and ruled out 83% of those without. The NPVs were generally high for the mentioned cutoffs (≥94%) whereas the PPVs were lower (≥56%), indicating a relatively large portion of false positives among the detected. With the exception of PPV, these values also correspond to an excellent overall accuracy (Table 3).

**Table 2:** Cases and noncases of anxiety and depression symptoms in patients with newly diagnosed breast, colorectal, or prostate cancer.

Measures		Statistics			Cronbach alpha
		n (%)	Mean (SD)	Range	
**State Trait Anxiety Inventory-State** **(n=550)**		—^a^	34.8 (11.1)	20-73	.95
	Non-cases (<40)	385 (70.0)	—	—	—
	Cases (>39)	165 (30.0)	—	—	—
**HADS^b^** **A (n=558)**		—	5 (8.7)	0-18	.88
	0-7	432 (77.4)	—	—	—
	8-10	73 (13.1)	—	—	—
	11-21	53 (9.5)	—	—	—
**eVAS^c^** **A (n=555)**		—	12.2 (18.8)	—	—
	0-20	451 (81.3)	—	—	—
	21-40	48 (8.6)	—	—	—
	41-60	29 (5.2)	—	—	—
	61-80	24 (4.3)	—	—	—
	81-100	3 (0.6)	—	—	—
**Montgomery Åsberg Depression Rating Scale-Self-report** **(n=551)**		—	7 (7)	0-37	.88
	Non-cases (<13)	444 (80.6)	—	—	—
	Cases (>12)	107 (19.4)	—	—	—
**HADS D (n=558)**		—	3.7 (3.4)	0-18	.85
	0-7	479 (85.9)	—	—	—
	8-10	52 (9.3)	—	—	—
	11-21	27 (4.8)	—	—	—
**eVAS D (n=552)**		—	10.2 (17.3)	0-100	—
	0-20	457 (82.8)	—	—	—
	21-40	52 (9.4)	—	—	—
	41-60	26 (4.7)	—	—	—
	61-80	14 (2.5)	—	—	—
	81-100	3 (0.6)	—	—	—

^a^Not Applicable.

^b^HADS: Hospital Anxiety and Depression Scale.

^c^eVAS: electronic Visual Analogue Scale.

**Table 3:** Results for different cutoff scores for anxiety- and depression-screening indices, with STAI-S^a^ >39 as a reference for anxiety and MADRS-S^b^ >12 as a reference for depression.

Measures	Cutoff score (>)	Sensitivity (%)	Specificity (%)	Positive predictive value (%)	Negative predictive value (%)
**HADS^c^** **anxiety (0-21)**					
	4	97	60	59	97
	5	95	71	67	94
	6	88	81	75	90
	7	77	89	87	86
**eVAS^d^** **anxiety (0-100 hs^e^)**					
	4	91	71	61	94
	5	89	76	63	93
	6	87	78	68	93
	7	86	83	70	93
	8	85	85	72	92
	9	82	86	73	91
	10	79	87	75	90
**HADS depression (0-21)**					
	4	97	72	56	97
	5	91	83	66	97
	6	87	89	76	94
	7	73	94	81	91
**eVAS depression (0-100 hs)**					
	4	90	71	48	96
	5	88	77	51	96
	6	85	80	54	96
	7	85	83	56	95
	8	83	84	58	95
	9	82	85	59	95
	10	80	86	60	94

^a^STAI-S: State Trait Anxiety Inventory-State.

^b^MADRS-S: Montgomery Åsberg Depression Rating Scale-Self-report.

^c^HADS: Hospital Anxiety and Depression scale.

^d^eVAS: electronic Visual Analogue Scale.

^e^hs: hundredths.

## Discussion

### Principal Findings

Web-based versions of eVAS and HADS were excellent with regard to the ability to discriminate between persons with and without clinical levels of anxiety or depression symptoms compared with Web-based versions of MARS-S and STAI-SS. Cutoffs of 5 or 6 on the HADS scales and 7 to 8 hs on the eVAS scales identified patients with anxiety or depression symptoms with high accuracy. Thus, Web-based screening with ultrashort and/or short questionnaires, instead of with longer more burdensome questionnaires, may be sufficient to identify patients with a need for psychosocial support.

### Prevalence of Depression and Anxiety

The prevalence of depression and anxiety in this sample is corroborated by studies in similar populations [1,2,47]. This indicates that Web-based screening of anxiety and depression performs equally compared with screening with paper-based questionnaires and may thereby be an adequate screening method in a clinical setting. However, treatment-related consequences may influence the prevalence. A recent review showed that the prevalence of depression among women with breast cancer who had received chemotherapy was higher than among the patients who did not. The risk for depression was higher during the first year after diagnosis, and those receiving adjuvant chemotherapy had higher levels of depression than those who did not. In addition, adverse symptoms of the treatment were associated with decreased health-related quality of life and increased levels of depression [48]. In this study, a majority were under treatment and newly diagnosed, which may have increased anxiety and depression symptoms even though the levels are relatively low.

### Determination of Optimal Cutoff Scores of Anxiety and Depression Indices

A questionnaire’s ability to detect as many patients with symptoms as possible and to miss as few as possible is of major importance in a first-step screening process. This ability usually comes with the tradeoff of a higher proportion of false positives that must be further evaluated in a more in-depth assessment, which in turn adds extra costs to an already strained health care sector [49]. Thus, there is a delicate balance between an excessively high and excessively low cutoff score from a clinical point of view.

#### Anxiety

For HADS anxiety, a cutoff of >5 may be suggested as it has the highest ability to correctly identify a patient with clinical symptoms of anxiety, weighing in both sensitivity and specificity. Previous Swedish studies in similar settings found that a cutoff of >4 on HADS anxiety was the best to detect the patients who deteriorated in their psychological health and quality of life during the disease trajectory [32,50]. However, as declared in the method section, there is still no consensus regarding the optimal cutoff for any of the HADS subscales for the paper-based format, indicating that future evaluations of the Web-based version of HADS will also yield various recommendations regarding cutoff scores. A cutoff score of >7 hs on eVAS anxiety may be recommended as this score has a high sensitivity, according to our results. In a previous comparison study of the eVAS anxiety and STAI-S, it was found that the 1-item eVAS could be readily completed and that it adequately measured anxiety [51]. Yet, further evaluations of the eVAS are needed before firm conclusions regarding an optimal cutoff can be drawn.

#### Depression

As an initial step in a screening procedure, a cutoff score of >5 on HADS depression may be preferable as it has a high ability to identify a patient with clinical symptoms of depression as well as to avoid failure detecting individuals without symptoms of depression. The HADS is the most thoroughly evaluated measurement of depression in oncology settings but there are difficulties comparing findings with earlier studies as many cutoffs have been used. Even lower cutoffs have been recommended for patients with cancer [31,52], and consensus about the optimal cutoff is still not reached. Regarding the eVAS, a cutoff score of >7 hs may be most suitable as it has a high ability to identify a patient with depression symptoms and rule out the ones without, but again, this need to be further evaluated.

### Strength and Limitations

This study has some limitations. First, the sample was restricted to newly diagnosed patients with breast, prostate, or colorectal cancer or a colorectal cancer relapse. Thus, the results may not represent patients with other cancer diagnoses or long-term survivors. On the contrary, we have studied a group of both curable and noncurable patients, which increases the probability that the results could be generalized to a bigger group than the one in this study. Another limitation is that the data are collected within a longitudinal intervention study that is much more demanding than a study where the participants are asked to answer questionnaires at a single observation point. This may have affected the sample and the number of nonparticipants and withdrawals (n=939). Our results need to be confirmed by future studies designed to compare Web-based instruments. In addition, we did not include patients who were not internet users and participants who declined Web-based studies, commonly representing an important group of older, sicker, and less-educated patients [53].

There may be a problem with face validity with regard to the Web-based version of the eVAS in this study as the mean score was low compared with the paper and pen versions [23]. However, mean ratings for eVAS have been shown to be equal when comparing the value received from eVAS with the one gathered via other formats in Web-based studies [54]. Another explanation of the low scores on the eVAS could be the wording of the questions. In this study, we used the expressions *anxiety* and *depression*, which may have been interpreted as a relatively severe condition compared with *worry* and *low mood*, which are more commonly used in Swedish. This choice of wording may have led to a higher number of true positives but also to more undetected patients with symptoms. This needs to be further elaborated upon to enable any certain conclusions.

We used the MADRS-S and STAI-S as reference instruments for depression and anxiety, which is not beyond criticism, given that the MADRS-S and STAI-S are not the most validated instruments in cancer settings. However, they have both shown good psychometric properties in various studies in different settings, including cancer and Web-based settings [40,55,56]. The ability to detect patients at suicidal risk is documented in the Web-based version of MADRS-S [40], which is of major importance in Web-based screening. The choice of self-report measures to establish the expected prevalence of anxiety and depression as well as relevant cutoffs should be interpreted with caution, whereas in-depth clinical interviews would have been more reliable.

The internal consistency was good in all 4 instruments indicated by high Cronbach alpha values. The sample size was good given that 558 patients responded to the questionnaires.

### Clinical Implications

This study indicates that the ultrashort and short eVAS and HADS may be used in a Web-based screening procedure in cancer care. Thus, a short screening instrument for anxiety and depression may be suggested in the initial step of a screening process, as they are less demanding and time-consuming. However, only 37.3% (558/1497) of eligible patients completed the Web-based questionnaires in this study. One of the main reasons for this was a lack of internet access. This makes it clear that Web-based screening strategies must include easy access to touch screens or tablets and support for the use of such devices. Both the eVAS and HADS perform well with regard to the ability to identify a majority of patients with anxiety and depression symptoms. The problem with false positives can be solved by further Web-based assessments of the patients reporting symptoms to allocate the support adequately. Some studies recommend the HADS as a follow-up instrument to detect cases after initial screenings with an ultrashort instrument [57], whereas others demonstrate that the HADS is more suitable as an instrument for initial screenings of depression and anxiety and that it cannot be recommended as a diagnostic (case-finding) instrument [17,58]. Patients reporting symptoms in the initial screening process may be further assessed with instruments with a higher specificity and must always be followed by a clinical assessment for diagnostics and treatment decisions.

### Suggestions for Future Research

The existing Web-based instruments are often paper questionnaires that have been adapted for Web-based use. The correlation between paper and Web-based versions of the same instruments correlate strongly, but differences such as mean scores and psychometrics do appear [22]. Further research on the Web-based versions is crucial to evaluate the reliability and validity of the existing instruments. Development of a new Web-based screening instrument may be optimal and should be considered in future research. This study has contributed to the field by demonstrating that the eVAS and HADS are comparable with longer questionnaires with regard to identification of patients with anxiety and depression symptoms and may be used as an initial Web-based screening instrument among patients with cancer. However, screening in itself is not sufficient. It has to take place in an infrastructure comprising adequate support and follow-up, and studies regarding the prerequisite for successful implementation in a clinical setting are needed.

### Conclusions

The use of the short and ultrashort tools eVAS and HADS is a suitable initial method of Web-based screening in busy clinical settings. However, there are still a large proportion of patients who lack access to the internet or the ability to use it. There is a need for special solutions for this group to find all the patients with psychological distress.

## Abbreviations

AUC: area under the curve
eVAS: electronic Visual Analogue Scale
HADS: Hospital Anxiety and Depression Scale
MADRS-S: Montgomery Åsberg Depression Rating Scale-Self-report
NPV: negative predictive value
PPV: positive predictive value
ROC: receiver operating curve
STAI-S: State Trait Anxiety Inventory-State
VAS: Visual Analogue Scale
